# Genetic Polymorphisms in *miR-604*A>G, *miR-938*G>A, *miR-1302-3*C>T and the Risk of Idiopathic Recurrent Pregnancy Loss

**DOI:** 10.3390/ijms22116127

**Published:** 2021-06-07

**Authors:** Sung-Hwan Cho, Ji-Hyang Kim, Hui-Jeong An, Young-Ran Kim, Eun-Hee Ahn, Jung-Ryeol Lee, Jung-Oh Kim, Jung-Jae Ko, Nam-Keun Kim

**Affiliations:** 1 Department of Biomedical Science, College of Life Science, CHA University, Seongnam 13488, Korea; arana006@naver.com (S.-H.C.); tody2209@naver.com (H.-J.A.); jokim8505@gmail.com (J.-O.K.); highko@cha.ac.kr (J.-J.K.); 2 CHA Bundang Medical Center, Department of Obstetrics and Gynecology, School of Medicine, CHA University, Seongnam 13496, Korea; bin0902@chamc.co.kr (J.-H.K.); happyiran@cha.ac.kr (Y.-R.K.); bestob@cha.ac.kr (E.-H.A.); 3 College of Life Science, Gangneung-Wonju National University, 7 Jukheon-gil, Gangneung 25457, Korea; 4 Department of Obstetrics and Gynecology, Seoul National University Bundang Hospital, Seongnam 13488, Korea; leejrmd@snu.ac.kr

**Keywords:** recurrent pregnancy loss, single nucleotide polymorphism (SNP), microRNA, MTHFR, GnRHR

## Abstract

The purpose of this study was to investigate whether polymorphisms in five microRNAs (miRNAs), *miR-604*A>G, *miR-608*C>G, *631*I/D, *miR-938*G>A, and *miR-1302-3*C>T, are associated with the risk of idiopathic recurrent pregnancy loss (RPL). Blood samples were collected from 388 patients with idiopathic RPL (at least two consecutive spontaneous abortions) and 227 control participants. We found the *miR-604* AG and AG + GG genotypes of *miR-604*, the *miR-938* GA and GA + AA genotypes of *miR-938*, and the *miR-1302-3*CT and CT + TT genotypes of *miR-1302-3* are less frequent than the wild-type (WT) genotypes, *miR-604*AA, *miR-938*GG, and *miR-1302-3*CC, respectively, in RPL patients. Using allele-combination multifactor dimensionality reduction (MDR) analysis, we found that eight haplotypes conferred by the miR-604/miR-608/miR-631/miR-938/miR-1302-3 allele combination, A-C-I-G-T, A-C-I-A-C, G-C-I-G-C, G-C-I-G-T, G-G-I-G-C, G-G-I-G-T, G-G-I-A-C, G-G-D-G-C, three from the *miR-604/miR-631/miR-938/miR-1302-3* allele combination, A-I-G-T, G-I-G-C, G-I-A-T, one from the *miR-604/miR-631/miR-1302-3* allele combination, G-I-C, and two from the *miR-604/miR-1302-3* allele combination, G-C and G-T, were less frequent in RPL patients, suggesting protective effects (all *p* < 0.05). We also identified the *miR-604*A>G and *miR-938*G>A polymorphisms within the seed sequence of the mature miRNAs and aligned the seed sequences with the 3′UTR of putative target genes, methylenetetrahydrofolate reductase (*MTHFR*) and gonadotropin-releasing hormone receptor (*GnRHR*), respectively. We further found that the binding affinities between *miR-604/miR-938* and the 3′UTR of their respective target genes (*MTHFR, GnRHR*) were significantly different for the common (*miR-604*A, *miR-938*G) and variant alleles (*miR-604*G, *miR-938*A). These results reveal a significant association between the *miR-604*A>G and *miR-938*G>A polymorphisms and idiopathic RPL and suggest that miRNAs can affect RPL in Korean women.

## 1. Introduction

Recurrent pregnancy loss (RPL) is usually defined as three or more consecutive pregnancy losses before the gestational age of 20 weeks. However, the American Society for Reproductive Medicine has recently redefined this condition as two or more consecutive pregnancy losses [1,2]. Pregnancy loss occurs in one of eight pregnant women, most often within 2–3 months of conception [3], and the likelihood of pregnancy loss is 5% higher for women who suffered a miscarriage during their first pregnancy than for healthy subjects [4]. The etiology of RPL includes several factors, such as genetics, anatomical deformities, endocrine dysfunction, placental anomaly, infection, smoking, excessive alcohol consumption, environmental factors, psychological trauma, and stress [5].

To date, increasing evidence has shown that deregulated miRNA expression underlies a wide variety of common reproductive disorders, ranging from implantation failure to endometriosis [6]. MicroRNAs (miRNAs) are small noncoding double-stranded RNAs (dsRNAs) of approximately 23 nucleotides (nt) that regulate their target genes through RNA silencing and the post-transcriptional regulation of gene expression [7]. These have been implicated in the regulation of several important biochemical pathways in eukaryotic organisms [8,9]. MiRNAs are initially transcribed as precursor pri-miRNAs by RNA polymerase II [10], which are converted to pre-miRNAs by the RNase III family enzyme DROSHA in a complex with the DiGeorge syndrome critical region 8 (DGCR8) protein [11]. The pre-miRNA is then exported to the cytoplasm by the exportin5 (Exp5)-ras-related nuclear protein (RAN)-GTP complex [12]. RAN is a small GTP-binding protein of the RAS superfamily that is essential for the translocation of RNA and proteins through the nuclear pore complex. The Ran GTPase binds Exp5 and forms a nuclear heterotrimer with pre-miRNA, facilitating its export from the nucleus [13]. Once in the cytoplasm, the pre-miRNA undergoes an additional processing step by the RNase III, dsRNA-specific endoribonuclease (DICER), generating a mature miRNA. DICER also initiates the formation of the RNA-induced silencing complex (RISC) [14], which is responsible for gene silencing via RNA interference (RNAi). 

Polymorphisms in pre-miRNAs were first reported in 2005 [15], and several miRNA polymorphism association studies have subsequently been conducted to examine their possible roles in various biological conditions [16,17]. Aberrant miRNA expression has further been implicated in numerous disease states, and consequently, several miRNA-based therapies are under investigation [18,19]. Notably, recent studies have found associations between specific miRNAs and reproductive disorders, such as endometriosis, pre-eclampsia, and infertility [20]. Associations between several miRNAs, including miRNA machinery genes, and the risk of RPL have been reported [21,22,23]. In particular, gene polymorphisms *miR-146a*C>G (rs2910164), *miR-149*T>C (rs2292832), *miR-196a2*T>C (rs11614913), and *miR-499*A>G (rs3746444) are present in RPL patients [24]. *MiR-146a* regulates FAS expression by binding to FAS mRNA, thereby reducing apoptosis [25]. These findings suggest that miRNAs may be essential for the normal functioning of the reproductive system.

In this study, we investigated the possible genetic causes of RPL by examining miRNA nucleotide polymorphisms in individuals suffering from this condition, focusing on the miRNAs previously implicated in reproductive diseases [26,27]. Notably, miRNA regulatory effect is a heritable trait in humans and that a polymorphism of the microRNA genes contributes to the observed inter-individual differences [28,29]. 

Transforming growth factor (TGF-β) superfamily members, including TGF-β and its associated regulatory miRNAs, miR-938 and miR-631 [30], exert critical functions in female reproductive physiology [31]. Another miRNA, miR-604, putatively binds targets related to placenta retention [32]. *The miR-608*C>G (rs4919510) polymorphism has been associated with the regulation of interleukin (IL)-6 expression [33], and the expression of this cytokine was higher in mice who experienced recurrent spontaneous abortion than in those with a normal murine pregnancy [34]. Lastly, the miR-1302 family, which is derived from MER53 elements, comprises placental-specific miRNAs [35]. We therefore hypothesized that these five miRNAs might be associated with female reproductive disease, and to test this, we investigated the association between RPL and miRNA polymorphisms, *604*A>G, *608*C>G, *631*I/D, *938*C>T, and *1302-3*C>T, in a population of Korean women. 

## 2. Results

### 2.1. Patient Clinical Characteristics

The clinical characteristics of RPL patients and control subjects enrolled in this study are summarized in Table 1. The mean ages of RPL patients and controls were 33.21 ± 4.55 and 33.37 ± 5.74 years, respectively, and both populations were 100% female. The clinical characteristics of both groups, including body mass index (BMI), number of previous pregnancy losses, mean gestational age, percent CD56+ natural killer (NK) cells, and prothrombin time (PT), as well as levels of homocysteine, folate, total cholesterol, uric acid, and plasminogen activator inhibitor (PAI)-1, are listed in Table 1. Table S2 shows the clinical variables of RPL patients stratified by miRNA polymorphism status; no significant difference was observed among RPL patients based on polymorphism status. However, in women with three or more pregnancy losses, uric acid levels were significantly higher in those with the *miR-608*CG genotype (*p* = 0.012) and PAI-1 levels were significantly higher in those with the *miR-1302-3*CT genotype (*p* = 0.004) compared with women with the respective wild-type (WT) homozygous genotypes (Table S3). Because abnormal plasma folate and homocysteine levels are known risk factors for pregnancy loss, we grouped the measurements of folate and homocysteine levels in RPL patients with two or more vs. three or more pregnancy losses by miRNA polymorphism status. From this analysis, we found that in women with three or more pregnancy losses, homocysteine levels were significantly higher in those with the *miR-938*G>A polymorphism, and folate levels were significantly lower in those with the *miR-1302-3*C>T polymorphism, than in women with the respective WT homozygous genotypes (Table S4).

### 2.2. Genetic Analysis

Table 2 shows the distribution of genotypes in RPL patients with two or more and three or more pregnancy losses and control subjects. The miRNA genotype frequencies of controls were consistent with the expected Hardy–Weinberg equilibrium values. Four combinations of miR-604A>G genotypes, two combinations of *miR-938*G>A genotypes, and two combinations of *miR-1302*-*3*C>T genotypes appeared to be protective vs. the respective WT homozygous genotypes, *miR-604*AA, *miR-938*GG, and *miR*-*1302-3*CC, in women with two or more pregnancy losses. Specifically, these were the *miR-604* genotypes AG (*p* = 0.016), GG (*p* = 0.008), AA vs. AG + GG (*p* = 0.005), and AA + AG vs. GG (*p* = 0.046), the *miR-938* genotypes GA and GG vs. GA + AA (*p* = 0.035 each), and the *miR*-*1302-3* genotypes CT and CC vs. CT + TT (*p* = 0.037 each). In women with three or more pregnancy losses, the *miR-604* genotypes, GG (*p* = 0.040) and AA vs. AG + GG (*p* = 0.033), the *miR-938* genotypes, GA and GG vs. GA + AA (*p* = 0.026 each), and the *miR*-*1302-3* genotypes, CT (*p* = 0.035) and CC vs. CT + TT (*p* = 0.027), were also protective against additional pregnancy losses. The frequencies of the *miR-608*C>G and *miR-631*I/D polymorphisms were not significantly different between RPL patients and control subjects.

We then carried out allele-combination multifactor dimensionality reduction (MDR) analysis. Among the haplotypes analyzed, 14 were less frequent in patients with RPL than in the controls, and these reduced frequencies were associated with lower susceptibility to RPL (Table 3). These include, from the analysis of all five miRNA loci (Figure S1) (*miR-604/miR-608/miR-631/miR-938/miR-1302-3*), the haplotypes A-C-I-G-T, A-C-I-A-C, G-C-I-G-C, G-C-I-G-T, G-G-I-G-C, G-G-I-G-T, G-G-I-A-C, and G-G-D-G-C; from the analysis of four miRNA loci (*miR-604/miR-631/miR-938/miR-1302-3*), the haplotypes A-I-G-T, G-I-G-C, and G-I-A-T; from the analysis of three loci (*miR-604/miR-631/miR-1302-3*), the haplotype G-I-C; and from analysis of two miRNA loci (*miR-604/miR-1302-3*), the G-C and G-T haplotypes. Only one haplotype (G-C-I-G-T) was more frequent in RPL patients (Table 3). 

Table 4 shows the results of the combined genotype analysis. Eight genotypes were less frequent in patients with RPL than in controls, and these reduced frequencies were associated with lower susceptibility to RPL. Specifically, these include the *miR-604*/*miR-608* combination AG + GG/CG + GG genotype, the *miR-604*/*miR-631* combination AG + GG/II and AG + GG/ID + DD genotypes, the *miR-604*/*miR-938* combination AG + GG/GG and AG + GG/GA + GA genotypes, the *miR-604*/*miR-1302-3* combination AG + GG/CC genotype, the *miR-604*/*miR-938* combination CG + GG/GA + AA genotype, and the *miR-631*/*miR-938* combination II/GA + AA genotype. 

### 2.3. 3′-UTR Target Gene Regulation by the miR-938G>A and miR-604A>G Polymorphisms

**Figure 1:**
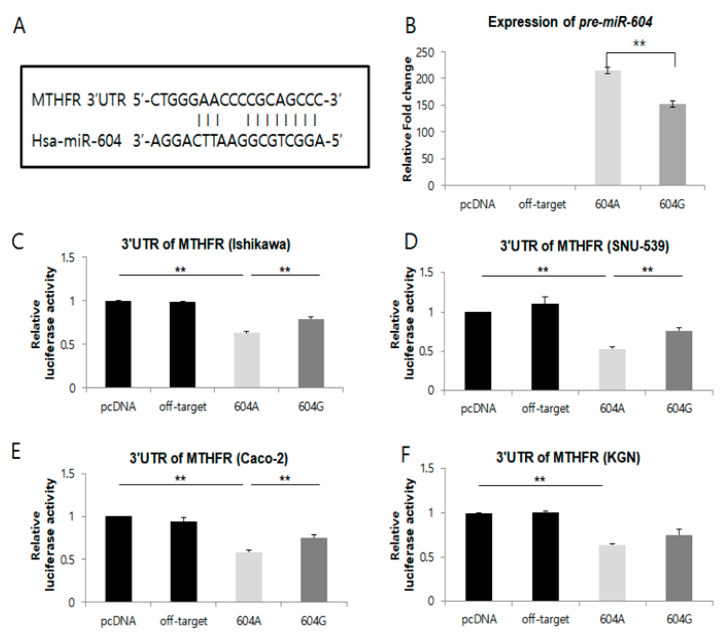
The microRNA, *miR-604,* regulates *MTHFR* mRNA via a targeting sequence located in the 3′UTR of this gene. (**A**) A schematic representation of putative target genes with 3′-UTRs that contain possible *miR-604*-A and *miR-604*-G binding sites in conserved regions. (**B**) Expression of *miR-604*A>G. Levels of *miR-604* were detected using microRNA RT-PCR in cells transfected with the empty pCR3.1 vector, pCR3.1-miR-offtarget, pCR3.1-*miR-604*-AA, or pCR3.1-*miR-604*-GG. U6 snRNA was used as an internal control, with the relative level of *miR-604* normalized to U6. ** *p* < 0.05. (**C**–**F**) Dual-luciferase reporter assays were performed to test the interaction of *hsa-miR-604* and its targeting sequence in the *MTHFR* 3′UTR using constructs containing the predicted targeting sequence (pGL4.13-*MTHFR* 3′UTR) cloned into the 3′UTR of the reporter gene. Data represent three independent experiments with triplicate measurements. ** indicates *p* < 0.05.

To investigate the functional impact of single nucleotide polymorphisms (SNPs) on the expression of *miR-938*G>A, *miR-604*A>G, and *miR-1302-3*C>T, we constructed *pri-miR-938*, *pri-miR-604*, and *pri-miR-1302-3* expression plasmids under the control of the CMV promoter with either the major or minor allele, and transfected the plasmids into a human endometrial cell line (Ishikawa, SNU-539), a human granulosa-like tumor cell line (KGN), a cancer cell line (Caco-2), and a gonadotropic pituitary cell line (GT1-7 for GnRHR). We found that the expression of *pre-miR-604* with the G allele was significantly lower than expression with the A allele (major) (*p* < 0.05) (Figure 1B). In addition, the expression of pre-miR-938 with the A allele was significantly lower than expression with the G allele (major) (*p* < 0.05) (Figure S2). However, the expression of pre-miR-1302-3 with the T allele was not significantly lower than with the C allele (major) (data not shown).

We then utilized genetic interaction analysis to predict miRNA target genes using TargetScanHuman (version 6.0, Whitehead Institute, Cambridge, MA, USA) software. Notably, the predicted target genes for each miRNA investigated included a reproduction-related protein. The *miR-604*G>A and *miR-938*G>A polymorphisms were also confirmed to be within the sequence of the pre-miRNA, which regulates methylenetetrahydrofolate reductase (*MTHFR*) and gonadotropin-releasing hormone receptor (*GnRHR*) expression by binding the mRNA, as shown in Table S5 and Figure 1 and Figure S2. 

We next assessed whether the allelic differences in regulatory activity observed for miR-604 and miR-938 could be attributed to differential binding to their target genes. To test this, we first co-transfected a plasmid containing the 3′UTR of the methylenetetrahydrofolate reductase (*MTHFR*) gene fused to a luciferase reporter gene construct and the pcDNA3.1 plasmid containing either the A or G allele of *miR-604* into Ishikawa, SNU-539, Caco-2, and KGN cells (Figure 1C–F). Compared with the vector control, we found that when the *MTHFR* reporter was co-transfected with *miR-604*, luciferase activity was significantly decreased in Ishikawa, SNU-539, Caco-2, and KGN cells (Figure 1C–F). Additionally, luciferase activity from the *MTHFR* reporter was significantly decreased in Ishikawa, SNU-539, and Caco-2 cells when co-transfected with the *miR-604* A allele (major), as compared to co-transfection with the miR-604 G (minor) allele (Figure 1C–E) (*p* < 0.05). Although this effect was not observed in KGN cells (Figure 1F). 

We also co-transfected a plasmid containing the 3′UTR of the *GnRHR* gene fused to a luciferase reporter gene construct and the pcDNA3.1 plasmid containing either the G or A allele of *miR-938* into GT1-7 cells (Figure S2). We found that the expression of *pre-miR-938* with the A allele (minor) was significantly lower than expression with the G allele (major) (*p* < 0.05) (Figure S2B). Further, compared with the *miR-938* A allele (minor), when the *GnRHR* reporter was co-transfected with the *miR-938* G allele (major), luciferase activity was significantly decreased in G allele (major) (*p* < 0.05) (Figure S2C). Collectively, these results suggest that the *miR-604*A>G and *miR-938* G>A polymorphism may alter the expression of their respective target genes (*MTHFR* and *GnRHR*) via differential binding to the 3′-UTR.

## 3. Discussion

Increasing evidence suggests that miRNAs play critical roles in several reproductive disorders [6,36,37]. Here, we investigated whether five pre-miRNA SNPs (*miR-604/-608/-631/-938/-1302*) are associated with the risk of RPL in a cohort of Korean women. From this analysis, we found that the polymorphic genotypes *miR-604*AG*, miR-604*GG*, miR-938*GA, and *miR-1302-3*CT are associated with a reduced risk of idiopathic RPL. Using genotype-based MDR analysis, we further found that eight haplotypes conferred by the *miR-604/miR-608/miR-631/miR-938/miR-1302-3* allele combination, A-C-I-G-T, A-C-I-A-C, G-C-I-G-C, G-C-I-G-T, G-G-I-G-C, G-G-I-G-T, G-G-I-A-C, G-G-D-G-C, three from the *miR-604/miR-631/miR-938/miR-1302-3* allele combination, A-I-G-T, G-I-G-C, G-I-A-T, one from the *miR-604/miR-631/miR-1302-3* allele combination, G-I-C, and two from the *miR-604/miR-1302-3* allele combination, G-C and G-T, were less frequent in RPL patients, suggesting that they may have protective effects (*p* < 0.05). 

In conditions with complex etiologies, such as RPL, the activities of many genes are interconnected, and thus gene–gene interactions may affect gene–disease associations. The MDR method is therefore valuable, as it can detect gene–gene interaction regardless of their chromosomal locations [38]. We further used a novel genotype based MDR approach to examine the potential interactive effects of different miRNAs on RPL. This analysis, which examined the effect of five miRNA polymorphisms on RPL, also suggested that gene–gene interactions between these five miRNA polymorphisms may play a role in this condition.

SNPs in miRNA genes, in genes encoding miRNA machinery proteins, or in miRNAs that target genes involved in miRNA synthesis or function will affect processes regulated by miRNAs and can adversely affect downstream gene expression [39,40]. Several studies have provided evidence supporting a critical role for miRNAs in RPL [41,42,43]. Here, we investigated whether specific polymorphisms in miRNAs linked to reproductive function are associated with RPL and whether these can affect binding to the 3′UTRs of their target gene and lead to allele-specific target gene regulation. It was previously shown that *miR-938* is associated with the TGF-β signaling pathway [30], and several TGF-β superfamily members exert critical functions in the female reproductive system. Specifically, these proteins regulate all aspects of ovarian follicle development, including primordial follicle recruitment, granulosa and theca cell proliferation, gonadotropin receptor expression, oocyte maturation, ovulation, luteinization, and corpus luteum formation [31,44]. In addition, prostaglandin F2 is required for placenta retention [32], and the 3′UTR of prostaglandin F2 receptor inhibitor contains a predicted binding target for *miR-604* (http://www.targetscan.org (accessed on 14 May 2017)). The *miR-608*C>G (rs4919510) polymorphism is associated with regulation of IL-6 expression [33], and previous reports have detected abnormal expression of IL-6 in recurrent spontaneous abortion compared with healthy individuals and animal models of normal pregnancy [34,45]. The 3′UTR of the *TGF-β3* gene contains a putative binding site for *miR-631*I/D (rs5745925) (http://www.targetscan.org (accessed on 14 May 2017)), a miRNA that targets the drug metabolism gene, *SULT1A1* [46], and the *miR-1302-3*C>T (rs7589328) polymorphism, which is derived from MER53 elements (a single transposon), are placental-specific miRNAs. The *hsa-miR-1302* family, found only in placental mammals, may comprise a placental-specific gene family [35]. 

We found that the expression of both pre-miR-604 and mature *miR-604* with the G allele was significantly lower (*p* < 0.05) than that of *miR-604* with the A allele (Figure 1B). These results suggest that the polymorphisms in *miR-604* could change expression of their target gene. 

An online search for *miR-604* and *miR-938* targets in the TargetScan and miRIAD (intragenic microRNA database) (http://bmi.ana.med. uni-muenchen.de/miriad/ (accessed on 14 May 2017)) provided a large number of putative mRNA targets. Among these, we focused on *MTHFR* for further functional analysis of *miR-604*A>G (Figure 1), as this gene is known to play several important roles in pregnancy and infertility [47,48]. In one-carbon metabolism, 5,10-methylenetetrahydrofolate (5,10-MTHF) is converted into 5-MTHF via the action of *MTHFR*. Methionine synthase then uses 5-MTHF as a methyl donor to convert homocysteine into methionine, resulting in the formation of tetrahydrofolate (THF). The missense mutation (Ala to Val resulting from a C to T mutation at 677 bp) in the *MTHFR* gene is a common mutation associated with deleterious effects on plasma homocysteine metabolism, leading to hyperhomocysteinemia and low folate levels [49]. There is a significant association between the *MTHFR* 677C>T mutation and unexplained RPL in the East Asian population [50]. According to a published report, an accumulation of homocysteine disrupts the proper response to the methionine cycle, leading to hyperhomocysteinemia, which has been correlated with an increased occurrence of blood clots, heart attacks, and strokes, as well as with early pregnancy loss [48]. 

A reporter construct containing the 3′UTR of *MTHFR* fused to a luciferase reporter was used to measure binding of variant and major alleles of miR-604G>A. From this analysis, we found that the binding affinity of miR-604 was stronger in cell lines transfected with the major A allele compared with those transfected with the minor G allele. Our data, therefore, suggest that the expression of *miR-604*A>G can influence the binding affinity of this miRNA to the 3′-UTR of *MTHFR*. Consistent with this observation, our results further show that expression levels from the *MTHFR* reporter are higher in cells expressing the minor G-type allele of miR-604 than in those with the major A-type allele. Based on these data, we speculate that the deregulation of *MTHFR* expression mediated by the A to G substitution in *miR-604* (rs2368393) may exert an influence on one-carbon metabolism. However, the allelic differences in *miR-604*A>G expression cannot be considered consistent with the regulation activity of *miR-604.*

One-carbon metabolism is associated with defects in blood coagulation factors, abortion, abnormal plasma urate, folate, and homocysteine levels, all of which are risk factors for vascular disease and RPL. Our results suggest that the *miR-604*A>G polymorphism and its effect on *MTHFR* might contribute to RPL and as such, should be considered when evaluating RPL patients. However, we did not test the binding of miRNAs to their target mRNAs by an alternative and more direct method. There have been few association studies in this area [51,52], and the results of the present study do not conclusively establish the significance of these polymorphisms in RPL. Further investigations of this and other pre-miRNA polymorphisms in diverse ethnic populations combined with functional studies will aid in the understanding of the role of miRNA polymorphisms in RPL and RPL susceptibility. 

We also searched for *miR-938* targets using the TargetScan and miRIAD databases and found a large number of putative candidates. Among these, we focused on *GnRHR* for the functional study of *miR-938*G>A (Figure S2). This gene, which encodes a receptor for the type 1 gonadotropin-releasing hormone, is essential for the proper development of the female reproductive system, and *GnRHR* function has been key to developing clinical strategies to treat various reproductive-related disorders [53,54]. The *miR-938*G>A polymorphism has been associated with the cytokine TGF-β, which induces a variety of cellular responses through a central signaling pathway. The down-regulation of TGF-β signaling through a target gene that is regulated by *miR-938* may have a possible role in the regulation of signaling pathways involved in the RPL [30]. TGF-β binds Type II receptors, in turn heterodimerize with Type I receptors, activating the receptor’s serine/threonine kinase activity and ultimately activating target genes in the nucleus. TGF-β1 also inhibits the secretion and activity of many other cytokines, including interferon (IFN)-γ, tumor necrosis factor (TNF)-α, and various interleukins. The association of *miRNA-938* with *GnRHR* expression can be considered in the context of the association between *GnRH* agonists and hypothyroidism. Specifically, hypothyroidism has been consistently shown to be associated with an increased risk for pregnancy complications [55], as well as detrimental effects on fetal neurocognitive development. Maternal hypothyroidism is also associated with increased risk of preterm birth, low birthweight, and loss of pregnancy [56]. Thyroid hormones affect the oocytes at the granulosa and luteal cell level [57], and it is thought that high prolactin levels alter the pulsatility of gonadotropin-releasing hormone and interfere with normal ovulation. 

To further explore the possible molecular mechanisms by which *miR-938*G>A affects gene expression, we analyzed the binding of *miR-938*G>A to the 3′UTR its target gene, *GnRHR*, using a luciferase reporter construct (Figure 1). We found that the binding of the common and variant alleles of *miR-938* to the 3′UTR of *GnRHR* was significantly different. That is, stronger binding was observed in cell lines transfected with *miR-938* containing the WT G allele compared with those transfected with the variant A allele. Our data, therefore, suggest that *miR-938*G>A can influence the binding of this miRNA to the 3′-UTR of *GnRHR*.

According to a published report, increased *GnRHR* expression in the immortalized gonadotropic cell line, LbT2, disrupts the response to follicle stimulating hormone (FSH) [58]. FSH is the primary gonadotropin responsible for pregnancy progression [59] and optimal levels of this hormone are extremely critical, as it helps to form the placenta, especially during the initial months of pregnancy [60]. Here, our clinical data show that FSH levels in subjects with the A type (variant) of *miR-938* are more than three times higher than FSH levels in those with the G-type allele (Table S1). We, therefore, speculate that the abnormal regulation of *GnRHR* resulting from G to A substitution in *miR-938* (rs12416605) may result in a disruption of the proper response to FSH and aberrant FSH expression. Notably, imbalances in the levels of homocysteine and folate are thought to contribute to lower birth weights [61], with higher homocysteine and lower folate concentrations in early pregnancy associated with lower placental weight and birthweight. However, from this analysis, we did not detect an association between folate and homocysteine concentrations and placental weight (Table 2 and Table S4). 

Our research has some limitations. First, we did not test the binding of miRNAs to the target gene by more direct methods. The mechanism by which polymorphisms in microRNAs affect the onset of RPL is unknown. Second, large data sets for mega-analysis should be collected, including information from other countries. Third, information regarding other risk factors for RPL patients is lacking, and a functional analysis of these factors is needed.

In summary, we found that the *miR-604*AG and AG + GG genotypes, the *miR-938*GA and GA + AA genotypes, and the *miR-1302-3*CT and CT + TT genotypes are less frequent than the WT genotypes, *miR-604*AA, *miR-938*GG, and *miR-1302-3*CC, in RPL patients. We also identified the *miR-938*G>A and *miR-604*A>G polymorphisms within the seed sequence of the mature miRNAs and aligned the seed sequences with the 3′UTR of their respective target genes (*GnRHR* and *MTHFR*). We then observed that the binding affinities between both *miR-938* and *miR-604* and the 3′UTRs of their putative target genes (*GnRHR* and *MTHFR,* respectively) were significantly different for miRNAs containing the major (*miR-938*G and *miR-604*A) and minor alleles (*miR-938*A and *miR-604*G). Several genetic studies of miRNAs using the candidate gene approach have now been reported, and a number of potentially causal miRNA polymorphisms have been identified in reproductive disease. However, to our knowledge, there are no other examples demonstrating how SNPs at the miRNA level affect the 3′-UTR of RPL-related target genes in vitro. Thus, we provide the first evidence that genetic polymorphisms in *miR-938* and *miR-604* modulate regulation of two RPL-related target genes. 

## 4. Materials and Methods

### 4.1. Ethics Statement

The study protocol was approved the study on 23 January 2010 (2010-01-123) by the Institutional Review Board of CHA Bundang Medical Center. All study subjects provided written informed consent prior to enrollment, and all methods applied in this study were carried out in accordance with the approved guidelines.

### 4.2. Study Subjects

Blood samples were collected from 388 patients with idiopathic RPL (mean age ± standard deviation [SD], 33.21 ± 4.55 years; mean BMI ± SD, 21.49 ± 3.84) and 227 control participants (mean age ± SD, 33.37 ± 5.74 years; mean BMI ± SD, 21.65 ± 3.44). All RPL patients were diagnosed based on at least two consecutive spontaneous abortions; the average gestational age and number of miscarriages were 7.36 ± 1.93 weeks and 3.28 ± 1.84 miscarriages, respectively. The RPL patients were assessed by analysis of human chorionic gonadotropin (hCG) levels, ultrasonography, and physical examination, before the gestational age of 20 weeks. No participant had a history of smoking or alcohol use. Patients with implantation failure due to anatomic, hormonal, chromosomal, infectious, autoimmune, or thrombosis-based causes were excluded from the study group. Potential anatomic causes were evaluated using hysterosalpingography, hysteroscopy, computerized tomographic scanning, and magnetic resonance imaging, to define intrauterine adhesions, septate uteri, and uterine fibroids. Hormonal causes of miscarriage, including hyperprolactinemia, luteal insufficiency, and thyroid disease, were evaluated by determining the blood levels of the appropriate hormones. To determine a chromosomal cause for miscarriage, chromosome analysis was performed using standard protocols [62], and metaphase chromosomes were stained using the GTG banding method. Infection with *Ureaplasma urealyticum* and *Mycoplasma hominis* was evaluated using bacterial culture. Autoimmune disease, defined as antiphospholipid syndrome and lupus, was evaluated using lupus anticoagulant and anticardiolipin antibodies. Thrombosis-based causes were defined as thrombophilia and were evaluated by the detection of protein C and protein S deficiencies and by the presence of anti-beta2 glycoprotein. RPL patients were enrolled between March 1999 and February 2010 in the Department of Obstetrics and Gynecology, and Fertility Center of CHA Bundang Medical Center, in Seongnam, Korea. Women in the control group were also recruited from CHA Bundang Medical Center and met the following criteria: pregnant, regular menstrual cycles, a history of at least one naturally conceived pregnancy, no history of pregnancy loss, and karyotype 46, XX.

### 4.3. Genotyping Analysis

DNA samples from RPL patients and controls were extracted using the G-DEX IIb Genomic DNA Extraction Kit-BLOOD (iNtRON Biotechnology Inc., Seongnam, Korea), as previously described [63]. Five miRNA SNPs were selected using the human genome SNP database (dbSNP, http://www.ncbi.nlm.nih.gov/snp (accessed on 28 January 2017)): *604*A>G (rs2368393), *608*C>G (rs4919510), *631*I/D (rs5745925), *938*C>T (rs12416605), and *1302-3*C>T (rs7589328). The location and host gene name of all miRNAs are listed in Table S6. The patient samples were genotyped using polymerase chain reaction-restriction fragment length polymorphism (PCR-RFLP) analysis. The *miR-604*A>G polymorphism was detected using the primers, (forward) 5′-CTT GGC TCA GTG GTC TGT TT-3′ and (reverse) 5′-GTA CAG GGA CTG AAA GGT GAA G-3′, under conditions of initial denaturation at 95 °C for 5 min, followed by 35 cycles of denaturation at 95 °C for 30 s, annealing at 59 °C for 30 s, and extension at 72 °C for 30 s, and a final extension at 72 °C for 5 min. The *miR-608*C>G polymorphism was detected using the primers, (forward) 5′-GTG GGT CAC ACT TGT AAT CT-3′ and (reverse) 5′-AAT TCT GAG GGT GTT CAC TG-3′, under conditions of initial denaturation at 95 °C for 15 min, 40 cycles of denaturation at 95 °C for 20 s, annealing at 60 °C for 40 s, and extension at 72 °C for 30 s, and a final extension at 72 °C for 5 min. The *miR-631*I/D polymorphism was detected using the primers, (forward) 5′-AAT CCC ACT CCA GGA TGG GAA A-3′ and (reverse) 5′-TGA CAG AGG AAC AGG CAG AGA T-3′, under conditions of initial denaturation at 95 °C for 5 min, 35 cycles of denaturation at 95 °C for 30 s, annealing at 61 °C for 30 s, and extension at 72 °C for 30 s, and a final extension at 72 °C for 5 min. The *miR-938*G>A polymorphism was detected using the primers, (forward) 5′-T GGT GCA CTG GGT TCA CCT TTA AGC G-3′ and (reverse) 5′-GTA ATA CCT CTG AGC CTT TGG GGC C-3′, under conditions of initial denaturation at 95 °C for 5 min, 35 cycles of denaturation at 95 °C for 30 s, annealing at 64 °C for 30 s, and extension at 72 °C for 30 s, and a final extension at 72 °C for 5 min. The *miR-1302-3*C>T polymorphism was detected using the primers, (forward) 5′-AAC TAA GCT TGG GAA ATA TTT ATG CCA-3′ and (reverse) 5′-GAG CAT CAT CAG TCC AAA GTC C-3′, under conditions of initial denaturation at 95 °C for 5 min, 35 cycles of denaturation at 95 °C for 30 s, annealing at 58 °C for 30 s, and extension at 72 °C for 30 s, and a final extension at 72 °C for 5 min. PCR products were digested using the following restriction enzymes: *Bss*SI*, Pvu*II*, Nla*IV*, Hha*I, and *Nla*III (New England BioLabs, Ipswich, MA, USA) (Table S7).

### 4.4. Clinical Characteristics of RPL Patients and Control Subjects

Plasma PAI-1, homocysteine, folic acid, total cholesterol, and uric acid were measured in blood collected from RPL patients after a 12-h fast. PAI-1 levels were determined using a human serpin E1/PAI-1 Quantikine ELISA Kit (R&D Systems, Minneapolis, MN, USA). Total cholesterol and uric acid were measured using a commercial enzymatic colorimetric test (Roche Diagnostics, Mannheim, Germany). Folic acid was measured using a competitive immunoassay with the ACS:180 (Bayer Diagnostics, Tarrytown, NY, USA). Homocysteine was measured using a fluorescence polarization immunoassay with the Abbott IMx analyzer (Abbott Laboratories, Abbott Park, IL, USA). To assess blood coagulation status, platelets (PLT) were counted using the Sysmex XE-2100 Automated Hematology System (Sysmex Corporation, Kobe, Japan). PT was measured with an ACL TOP automated photo-optical coagulometer (Beckman Coulter).

### 4.5. Expression Vectors Construction

To synthesize pri-miR-604 A, pri-miR-604 G, pri-miR-938 G, and pri-miR-938 A, genomic fragments (pri-miR-604, 416 bp; pri-miR-938, 522 bp) as well as pre-miR604 and pre-miR938 and their flanking regions were amplified from human genomic DNA with primer s (miR-604, forward 5′-AATAGACCAGGGCACCCTCT, reverse 5′-TGAAAGGTGAAGCCAATTCC; miR-938, forward 5′-AAGATTTGGCAGTGATCTTT, reverse 5′-AGGCCTGACTTCATAAGAAT) and cloned into the vector pcDNA3.1 (-) (Invitrogen, Carlsbad, CA, USA) with a XhoI (CCGCTCGAGCGG)/BamHI (CGGGATCCCG) linker. To create single-point mutations we used the Muta-Direct Site Directed Mutagenesis Kit (iNtRON Biotechnology Inc., Seoul, Korea). PCR primers for miR-604 were forward 5′-TGA CCT TCC ACG CTC GCG TGT CCA CTA GCA G and reverse 5′-CTG CTA GTG GAC ACG CGA GCG TGG AAG GTC A) and for miR-938 were forward 5′-GAA GGT GTA CCA TGT ACC CTT AAA GGT GAA, and reverse 5′-TTC ACC TTT AAG GGT ACA TGG TAC ACC TTC. The sequences of both constructs were confirmed by direct sequencing, and the only difference detected was in the SNP. To create target gene::luciferase reporters for miR-604 and miR-938, a region of the MTHFR target gene corresponding to the 3′UTR region (Origene, Rockville, MD, USA) and a region of the GnRHR gene, corresponding to the 3′UTR region (Origene, Rockville, MD, USA), were amplified and cloned into the pGL4.13-luciferase vector (Promega, Madison, WI, USA) [64,65]. The cDNAs were PCR amplified using the primers, forward 5′-GCA ATT GTG GGA TGT CCT CT-3′ and reverse 5′-GAG CTG TGT GTG CAG TTT GG-3′ (MTHFR), forward 5′-GCT CTA GAG CTG GCA CGT CCT TTC TTT CTT-3′ and reverse 5′-TTT GGC CGG CCA AAC AGT CTG GTC CAT CCC TCT C-3′ (GnRHR). All constructs were verified by sequencing.

### 4.6. Cell Transfection and Luciferase Assay

Cells from a human endometrial cell line (Ishikawa, SNU-539), a gonadotropic pituitary cell line (GT1-7) for GnRHR, a cancer cell line (Caco-2), and cells from a human granulosa (KGN) were plated at 1 × 10^6^ cells per well in 6-well plates and transfected 24 h later using JetPRIME transfection reagent (Polyplus, France). Transfection reactions for miR-604 contained 500 ng of 604-A (in pcDNA3.1-) or 500 ng of 604-G (in pcDNA3.1-) with 500 ng of 3′UTR-MTHFR in pGL4.13 and 200 ng of pGL4.75 (Renilla-normalization control), or, for miR-938, contained 500 ng of 938-C (in pcDNA3.1-) or 500 ng of 938-T (in pcDNA3.1-) with 500 ng of 3′UTR-GnRHR in pGL4.13 and 200 ng of pGL4.75 (Renilla-normalization control). As an additional control, we used an off-target control miRNA (QIAGEN, Hilden, Germany; 1027271). After transfection for 24 h, the growth medium was removed, and the cells were washed gently with PBS. Passive Lysis Buffer (100 μL/well; Promega) was added, and plates were rocked gently for 15 min at room temperature, and 10 μL of cell lysates were transferred into white, opaque 96-well plates (Falcon, 353296). Firefly and Renilla luciferase activity assays were performed sequentially for cell lysates in each well using the Dual-Luciferase Reporter Assay System (DLR assay system, Promega, Madison, WI, USA). At each luminescence reading, after the injector dispensed assay reagents into the well, there was a 2-s pre-measurement delay, followed by a 10-s measurement period. Luciferase assays were analyzed based on the ratio of Firefly/Renilla to normalize cell number and transfection efficiency. Cells co-transfected with 3′UTR-MTHFR or 3′UTR-GnRHR in pGL4.13 with the pGL4.73 vector served as a negative control [66,67].

### 4.7. Statistical Analysis

Differences in the frequencies of miRNA polymorphisms in control and patient groups were assessed using Fisher’s exact test and a logistic regression model. The odds ratios (ORs) adjusted OR (AORs), and 95% confidence intervals were calculated, and the mean and SD and percentages were determined. Data analysis was performed using MedCalc, v. 12.1.4 (MedCalc, Ostend, Belgium) and GraphPad Prism 4.0 (GraphPad, San Diego, CA, USA) software. The HAPSTAT program (v.3.0, www.bios.unc.edu/~lin/hapstat/ (accessed on 16 March 2017)) was used with a strong synergistic effect to estimate the frequency of polymorphic diploidy, with *p* < 0.05 considered as statistically significant. The false discovery rate (FDR) was used to adjust multiple comparisons, and an FDR-adjusted *p* value of <0.05 was considered statistically significant [68]. We used StatsDirect (Altrincham, UK) software to perform a regression analysis of miRNA polymorphism genotypes and risk factors. Genetic interaction analysis was performed with the open-source MDR software package (v.2.0) available from www.epistasis.org (accessed on 12 October 2017). The MDR method consists of two main steps [69,70]. We used genetic interaction analysis to predict miRNA target genes using TargetScanHuman (http://www.targetscan.org (accessed on 14 May 2017)).

## Figures and Tables

**Table 1:** Clinical characteristics of RPL patients and controls.

Characteristic	Controls (*n* = 227)	RPL Patients (*n* = 388)	p *
	Mean ± SD	Mean ± SD	
Age (years)	33.37 ± 5.74	33.21 ± 4.55	0.726
BMI (kg/m^2^)	21.65 ± 3.44	21.49 ± 3.84	0.533
Previous pregnancy losses (n)	None	3.28 ± 1.84	
RPL < 20 weeks (%)	None	98.90%	
Live births (n)	1.79 ± 0.74	None	
Mean gestational age (weeks)	39.29 ± 1.67	7.36 ± 1.93	<0.001
Homocysteine (μmol/L)	NA	6.98 ± 2.10	
Folate (mg/mL)	NA	14.21 ± 11.94	
Total cholesterol (mg/dL)	NA	187.73 ± 49.42	
Uric acid (mg/dL)	NA	3.80 ± 0.84	
CD56 + NK cells (%)	NA	18.26 ± 7.99	
PAI-1 (ng/mL)	NA	10.53 ± 5.72	
PT (sec)	NA	11.58 ± 0.86	

Abbreviations: RPL, recurrent pregnancy loss; SD, standard deviation; BMI, body mass index; NA, not applicable; PAI-1, plasminogen activator inhibitor-1; PT, prothrombin time. *p* *: *p*-values were calculated by chi-square test for categorical data and two-sided *t*-test for continuous data.

**Table 2:** Comparison of microRNA polymorphic genotype frequencies in controls and RPL patients.

Characteristics	Controls *n* = 227	PL ≥ 2 *n* = 388	AOR (95% CI) ^a^	*p* ^b^	FDR-*p* ^c^	PL ≥ 3 *n* = 206	AOR (95% CI) ^a^	*p* ^b^	FDR-*p* ^c^
	*n* (%)	*n* (%)				n (%)			
**miR-604A>G**									
AA	73 (32.2)	171 (44.1)	1.000 (reference)			86 (41.7)	1.000 (reference)		
AG	115 (50.7)	173 (44.6)	0.640 (0.445–0.920)	0.016	0.061	95 (46.1)	0.686 (0.452–1.04)	0.076	0.126
GG	39 (17.2)	44 (11.3)	**0.496 (0.296–0.832)**	**0.008**	**0.024**	25 (12.1)	**0.532 (0.292–0.970)**	**0.04**	0.12
Dominant (AA vs. AG + GG)			**0.606 (0.429–0.856)**	**0.005**	**0.025**		0.650 (0.438–0.965)	0.033	0.055
Recessive (AA + AG vs. GG)			0.621 (0.389–0.992)	0.046	0.138		0.646 (0.374–1.117)	0.118	0.276
**miR-608C>G**									
CC	48 (21.1)	93 (24.0)	1.000 (reference)			51 (24.8)	1.000 (reference)		
CG	109 (48.0)	189 (48.7)	0.885 (0.581–1.349)	0.57	0.57	103 (50.0)	0.867 (0.536–1.401)	0.559	0.559
GG	70 (30.8)	106 (27.3)	0.789 (0.497–1.252)	0.314	0.471	52 (25.2)	0.702 (0.411–1.199)	0.195	0.292
Dominant (CC vs. CG + GG)			0.850 (0.572–1.261)	0.419	0.419		0.805 (0.513–1.263)	0.345	0.345
Recessive (CC + CG vs. GG)			0.847 (0.591–1.214)	0.366	0.549		0.751 (0.492–1.146)	0.184	0.276
**miR-631I/D**									
II	204 (89.9)	357 (92.0)	1.000 (reference)			193 (93.7)	1.000 (reference)		
ID	23 (10.1)	31 (8.0)	0.778 (0.441–1.372)	0.385	0.481	13 (6.3)	0.577 (0.283–1.178)	0.131	0.163
DD	0 (0.0)	0 (0.0)	N/A	N/A	N/A	0 (0.0)	N/A	N/A	N/A
Dominant (II vs. ID + DD)			0.778 (0.441–1.372)	0.385	0.419		0.577 (0.283–1.178)	0.131	0.163
Recessive (II + ID vs. DD)			N/A	N/A	N/A		N/A	N/A	N/A
**miR-938G>A**									
GG	215 (94.7)	380 (97.9)	1.000 (reference)			204 (99.0)	1.000 (reference)		
GA	12 (5.3)	8 (2.1)	0.375 (0.151–0.933)	0.035	0.061	2 (1.0)	0.179 (0.040–0.811)	0.026	0.087
AA	0 (0.0)	0 (0.0)	N/A	N/A	N/A	0 (0.0)	N/A	N/A	N/A
Dominant (CC vs. CT + TT)			0.375 (0.151–0.933)	0.035	0.061		0.179 (0.040–0.811)	0.026	0.055
Recessive (CC + CT vs. TT)			N/A	N/A	N/A		N/A	N/A	N/A
**miR-1302-3C>T**									
CC	212 (84.1)	349 (89.9)	1.000 (reference)			188 (91.3)	1.000 (reference)		
CT	14 (15.5)	37 (9.5)	0.596 (0366–0.969 )	0.037	0.061	18 (8.7)	0.517 (0.280–0.955)	0.035	0.087
TT	1 (0.4)	2 (0.5)	1.101 (0.099–12.228)	0.937	0.937	0 (0.0)	N/A	0.994	0.994
Dominant (CC vs. CT + TT)			0.596 (0.366–0.969)	0.037	0.061		0.502 (0.273–0.952)	0.027	0.055
Recessive (CC + CT vs. TT)			1.177 (0.106–13.059)	0.894	0.894		N/A	0.994	0.994

Abbreviations: RPL, recurrent pregnancy loss; AOR, adjusted odds ratio; CI, confidence interval. ^a^ Adjusted for age. ^b^ Fisher’s exact test. ^c^ False-positive discovery rate (FDR)-adjusted *p*-value. Bold numbers indicate significant *p*-values. N/A: not applicable

**Table 3:** MDR-based allele-combination analysis of microRNA polymorphisms in idiopathic RPL patients and controls.

Allele Combination	Controls	RPL	AOR (95% CI) ^a^	*p* ^b^	FDR-*p* ^c^
	2*n* = 454	2*n* = 776			
	*n* (%)	*n* (%)			
**miR-604, miR-608, miR-631, miR-938, miR-1302-3**					
A-C-I-G-C	103 (22.7)	233 (30.1)	1.000 (reference)		
A-C-I-G-T	15 (3.3)	10 (1.3)	0.294 (0.128–0.678)	**0.004**	**0.025**
A-C-I-A-C	5 (1.2)	0 (0.0)	0.040 (0.002–0.736)	**0.003**	**0.025**
A-C-D-G-C	6 (1.4)	7 (1.0)	0.515 (0.169–1.573)	0.237	0.45
A-G-I-G-C	119 (26.2)	230 (29.6)	0.854 (0.620–1.177)	0.369	0.584
A-G-I-G-T	5 (1.1)	13 (1.7)	1.149 (0.399–3.309)	1.000	1.000
A-G-I-A-C	0 (0.0)	5 (0.6)	4.876 (0.266–89.06)	0.3278	0.566
A-G-I-A-T	0 (0.0)	1 (0.1)	1.33 (0.05–32.94)	1.000	1.000
A-G-D-G-C	7 (1.4)	15 (1.9)	0.947 (0.374–2.393)	1.000	1.000
A-G-D-G-T	0 (0.0)	1 (0.1)	1.33 (0.0536–32.94)	1.000	1.000
**G-C-I-G-C**	74 (16.3)	109 (14)	**0.651 (0.447–0.9479)**	**0.026**	0.081
G-C-I-G-T	0 (0.0)	10 (1.3)	9.308 (0.540–160.5)	0.036	0.086
G-C-I-A-C	0 (0.0)	3 (0.3)	3.103 (0.158–60.66)	0.556	0.704
G-G-D-G-C	0 (0.0)	3 (0.3)	3.103 (0.158–60.66)	0.556	0.704
G-G-I-G-C	90 (19.9)	125 (16.2)	0.614 (0.429–0.8772)	0.008	0.034
G-G-I-G-T	13 (2.8)	6 (0.8)	0.204 (0.075–0.5518)	**0.002**	**0.025**
G-G-I-A-C	4 (0.9)	0 (0.0)	0.049 (0.002–0.9238)	**0.009**	**0.034**
G-G-I-A-T	3 (0.5)	0 (0.0)	3.103 (0.158–60.66)	0.556	0.704
G-G-D-G-C	8 (1.9)	5 (0.7)	0.276 (0.088–0.8651)	0.03	0.081
G-G-D-G-T	2 (0.4)	0 (0.0)	0.088 (0.004–1.864)	0.095	0.2
**miR-604, miR-631, miR-938, miR-1302-3**					
A-I-G-C	219 (48.3)	460 (59.3)	1.000 (reference)		
A-I-G-T	22 (4.8)	24 (3.1)	0.5194 (0.2849–0.9469)	0.035	0.13
A-I-A-C	7 (1.6)	7 (0.9)	0.4761 (0.1649–1.374)	0.247	0.388
A-I-A-T	0 (0.0)	1 (0.1)	1.43 (0.05797–35.27)	1.000	1.000
A-D-G-C	12 (2.6)	22 (2.9)	0.87289 (0.4241–1.796)	0.71	0.867
A-D-G-T	0 (0.0)	1 (0.1)	1.43 (0.05797–35.27)	1.000	1.000
**G-I-G-C**	167 (36.9)	237 (30.6)	**0.6756 (0.5235–0.8721)**	**0.003**	**0.034**
G-I-G-T	11 (2.3)	15 (1.9)	0.6492 (0.2933–1.437)	0.291	0.401
G-I-A-C	2 (0.5)	1(0.1)	0.238 (0.02146–2.641)	0.246	0.388
G-I-A-T	3 (0.6)	0 (0)	0.06809 (0.003499–1.3250	0.034	0.13
G-D-G-C	9 (2.0)	8 (1.0)	0.4232 (0.1610–1.112)	0.112	0.247
G-D-G-T	2 (0.4)	0 (0.0)	0.09533 (0.004554–1.996)	0.105	0.247
**miR-604, miR-631, miR-1302-3**					
A-I-C	228 (50.1)	467 (60.2)	1.000 (reference)		
A-I-T	21(4.5)	25 (3.2)	0.5812 (0.3185–1.061)	0.078	0.161
A-D-C	12 (2.6)	22 (2.9)	0.8951 (0.4352–1.841)	0.851	0.993
A-D-T	0 (0.0)	1 (0.1)	1.466 (0.05945–36.16)	1.000	1.000
**G-I-C**	168 (37.1)	238 (30.6)	**0.6916 (0.5369–0.8909)**	**0.005**	**0.035**
G-I-T	14 (3.2)	15 (2.0)	0.5231 (0.2482–1.102)	0.106	0.161
G-D-C	9(2.1)	8 (1.0)	0.434 (0.1652–1.140)	0.115	0.161
G-D-T	2 (0.4)	0 (0)	0.09775 (0.004670–2.04)	0.108	0.161
**miR-604, miR-1302-3**					
A-C	239 (52.7)	489 (63)	1.000 (reference)		
A-T	21 (4.6)	26 (3.3)	0.6051 (0.3336–1.098)	0.110	0.110
**G-C**	178 (39.1)	246 (31.7)	**0.6755 (0.5275–0.8650)**	**0.002**	**0.007**
G-T	16 (3.6)	15(2.0)	0.4582 (0.2227–0.9426)	0.034	0.051

Abbreviations: RPL, recurrent pregnancy loss; AOR, adjusted odds ratio; CI, confidence interval; FDR-*p*, false discovery rate-adjusted *p*. MDR, multifactor dimensionality reduction. ^a^ Adjusted for age. ^b^ Fisher’s exact test. ^c^ False-positive discovery rate (FDR)-adjusted *p*-value. Bold numbers indicate significant *p*-values.

**Table 4:** Combined analysis of microRNA gene polymorphisms in RPL patients and controls.

Combination Genotype	Controls *n* = 227 *n* (%)	RPL *n* = 388 *n* (%)	AOR (95% CI) ^a^	*p* ^b^	FDR-*p* ^c^
miR-604A>G, miR-608C>G					
AA/CC	15 (6.6)	39 (10.1)	1.000 (reference)		
AA/CG + GG	58 (25.6)	132 (34.0)	0.870 (0.445–1.702)	0.684	0.846
AG + GG/CC	33 (14.5)	54 (13.9)	0.624 (0.298–1.305)	0.210	0.315
AG + GG/CG + GG	121 (53.3)	163 (42.0)	0.521 (0.275–0.990)	0.047	0.141
miR-604A>G, miR-631I>D					
AA/II	65 (28.6)	155 (39.9)	1.000 (reference)		
AA/ID + DD	8 (3.5)	16 (4.1)	0.841 (0.343–2.064)	0.705	0.846
**AG + GG/II**	139 (61.2)	202 (52.1)	0.611 (0.425–0.879)	**0.008**	**0.024**
AG + GG/ID + DD	15 (6.6)	15 (3.9)	0.432 (0.197–0.950)	0.037	0.056
miR-604A>G, miR-938G>A					
AA/GG	71 (31.3)	167 (43)	1.000 (reference)		
AA/GA + GA	2 (0.9)	4 (1.0)	0.852 (0.153–4.763)	0.856	0.856
AG + GG/GG	144 (63.4)	213 (54.9)	0.637 (0.448–0.905)	**0.012**	**0.018**
AG + GG/GA + GA	10 (4.4)	4 (1.0)	0.171 (0.052–0.565)	**0.004**	**0.012**
miR-604A>G, miR-1302-3C>T					
AA/CC	61 (26.8)	154 (39.6)	1.000 (reference)		
AA/CT + TT	11 (4.9)	17 (4.4)	0.617 (0.273–1.394)	0.506	0.846
AG + GG/CC	130 (57.3)	195 (50.3)	0.599 (0.412–0.864)	0.022	0.044
AG + GG/CT + TT	25 (11.0)	22 (5.7)	0.351 (0.182–0.673)	**0.006**	**0.018**
miR-608C>G, miR-938G>A					
CC/GG	48 (21.1)	91 (23.5)	1.000 (reference)		
CG + GG/GG	167 (73.6)	289 (74.5)	0.914 (0.613–1.361)	0.657	0.657
CG + GG/GA + AA	12 (5.3)	6 (1.5)	0.268 (0.095–0.762)	**0.014**	**0.021**
miR-631I>D, miR-938G>A					
II/GG	192 (84.6)	349 (89.9)	1.000 (reference)		
II/GA + AA	12 (5.3)	8 (2.1)	0.367 (0.147–0.914)	0.031	0.186
ID + DD/GG	23 (10.1)	31 (8)	0.752 (0.426–1.329)	0.327	0.382
miR-938G>A, miR-1302-3C>T					
GG/CC	182 (80.2)	342 (88.1)	1.000 (reference)		
GG/CT + TT	33 (14.5)	38 (9.8)	0.617 (0.374–1.017)	0.135	0.444
GA + AA/CC	9 (4.0)	7 (1.8)	0.371 (0.141–0.974)	0.210	0.077
GA + AA/CT + TT	3 (1.3)	1 (0.3)	0.351 (0.058–0.127)	0.481	0.694

Abbreviations: RPL, recurrent pregnancy loss; AOR, adjusted odds ratio; CI, confidence interval; FDR-*p*, false discovery rate-adjusted *p*; ^a^ Adjusted for age. ^b^ Fisher’s exact test. ^c^ False-positive discovery rate (FDR)-adjusted *p*-value. Bold numbers indicate significant *p*-values.

## Data Availability

The data presented in this study are available on request from the corresponding author.

## Supplementary Materials

Supplementary materials can be found at https://www.mdpi.com/article/10.3390/ijms22116127/s1.
