# Supplementary material for: Genetic Polymorphisms in miR-604A>G, miR-938G>A, miR-1302-3C>T and the Risk of Idiopathic Recurrent Pregnancy Loss
Source: Int J Mol Sci. 2021 Jun 7;22(11):6127. doi: 10.3390/ijms22116127 (PMC8201216; doi:10.3390/ijms22116127)
Supplement: Supplementary file 1 [file ijms-22-06127-s001.zip › ijms-1235002-supplementary.pdf]

Genetic Polymorphisms in *miR-604*A>G, *miR-938*G>A, *miR-1302-3*C>T and the Risk of Idiopathic Recurrent Pregnancy Loss

Sung Hwan Cho [1,†], Ji Hyang Kim [2,†], Hui Jeong An [1,3], Young Ran Kim [2], Eun Hee Ahn [2], Jung Ryeol Lee [4], Jung Oh Kim [1], Jung Jae Ko [1] and Nam Keun Kim [1,*]

[1] Department of Biomedical Science, College of Life Science, CHA University, Seongnam 13488, Korea; arana006@naver.com (S.H.C.); tody2209@naver.com (H.J.A.); jokim8505@gmail.com (J.O.K.); highko@cha.ac.kr (J.J.K.)

[2] Department of Obstetrics and Gynecology, CHA Bundang Medical Center, School of Medicine, CHA University, Seongnam 13496, Korea; bin0902@chamc.co.kr (J.H.K.); happyiran@cha.ac.kr (Y.R.K.); bestob@cha.ac.kr (E.H.A.)

[3] College of Life Science, Gangneung-Wonju National University, 7 Jukheon-gil, Gangneung, Gangwon 25457, Korea

[4] Department of Obstetrics and Gynecology, Seoul National University Bundang Hospital, Seongnam 13488, Korea; leejrmd@snu.ac.kr

* Correspondence: nkkim@cha.ac.kr; Tel.: +82-31-780-5762; Fax: +82-31-780-5766

† Sung Hwan Cho and Ji Hyang Kim contributed equally to this work.

Supplementary Table S1

FSH, LH and E2 levels of miRNA polymorphic genotypes in Control and RPL patients

Genotypes	FSH(mIU/mL)		LH(mIU/mL)		E2(pg/mL)	
	Control(113)	Case (388)	Control(113)	Case (388)	Control(113)	Case (388)
**miR-604A>G**						
AA	8.18 ± 10.48	7.17 ± 9.27	3.05 ± 4.08	35.10 ± 32.00	31.11 ± 283.40	5.29 ± 5.370
AG	8.26 ± 7.95	7.95 ± 12.29	3.36 ± 2.24	36.67 ± 28.49	22.88 ± 173.07	7.16 ± 16.61
GG	7.55 ± 4.53	6.91 ± 5.67	3.68 ± 3.36	33.34 ± 22.45	25.45 ± 166.34	7.95 ± 6.46
***P*[a]**	0.643	0.854	0.436	0.899	0.031	0.592
**miR-608C>G**						
CC	7.704 ± 4.10	5.75 ± 2.68	3.43 ± 1.96	4.33 ± 2.19	27.11 ± 13.92	40.07 ± 39.66
CG	7.93 ± 10.41	7.70 ± 11.59	3.17 ± 3.22	5.71 ± 7.11	28.04 ± 267.95	34.16 ± 26.92
GG	8.61 ± 7.50	8.80 ± 12.64	8.96 ± 9.93	9.26 ± 21.39	22.62 ± 174.45	34.67 ± 22.80
***P*[a]**	0.4	0.351	0.731	0.103	0.215	0.561
**miR-631I/D**						
II	8.15 ± 7.43	7.68 ± 10.95	3.30 ± 3.12	6.53 ± 12.61	27.27 ± 215.84	35.84 ± 30.25
ID	7.9 ± 12.93	5.80 ± 3.07	3.42 ± 2.70	3.79 ± 1.63	18.38 ± 169.96	34.36 ± 21.13
DD	NA	NA	NA	NA	NA	NA
p	0.743	0.351	0.796	0.373	0.025	0.849
**miR-938G>A**						
GG	8.16 ± 8.49	7.11 ± 8.97	3.36 ± 3.28	6.11 ± 11.84	25.43 ± 205.77	35.73 ± 29.54
GA	7.36 ± 1.36	27.03 ± 39.53	2.44 ± 0.75	15.16 ± 21.68	35.83 ± 381.45	34.2 ± 30.07
AA	NA	NA	NA	NA	NA	NA
***P*[a]**	0.743	< 0.001	0.25	0.139	0.093	0.929
**miR-1302-3C>T**						
CC	7.86 ± 7.16	7.77 ± 11.11	3.31 ± 3.28	6.52 ± 12.78	26.00 ± 226.10	35.30 ± 30.13
CT	10.05 ± 11.91	5.59 ± 3.26	3.33 ± 1.37	4.34 ± 3.45	25.93 ± 165.76	39.78 ± 21.70
TT	NA	NA	NA	NA	NA	NA
***P*[a]**	0.009	0.643	0.244	0.702	0.988	0.576

Abbreviations: RPL, recurrent pregnancy loss; FSH, Follicle-stimulating hormone; LH, Luteinizing hormone; E2, Estradiol; PLT, platelet count; SD, standard deviation.

[a]One-way analysis of variance test. [b]Mean ± SD.

Supplementary Table S2
Clinical variables in RPL patients, stratified by microRNA polymorphism status

Genotypes	CD56+ NK Cells (%) Mean ± SD	T. chol (mg/dl) Mean ± SD	PT (sec) Mean ± SD	PAI-1 (ng/ml) Mean ± SD	Uric acid (mg/dl) Mean ± SD	PLT (10^3/ul) Mean ± SD
**miR-604A>G**						
AA	17.608 ± 7.501	184.914 ± 49.466	11.643 ± 0.868	11.263 ± 5.961	3.728 ± 0.767	256.337 ± 66.487
AG	18.678 ± 8.503	189.543 ± 51.960	11.561 ± 0.865	10.172 ± 5.677	3.851 ± 0.917	253.168 ± 54.183
GG	18.467 ± 7.453	192.278 ± 37.644	11.400 ± 0.786	9.158 ± 4.889	3.894 ± 0.756	261.857 ± 49.529
P[a]	0.769	0.771	0.495	0.383	0.578	0.754
**miR-608C>G**						
CC	19.159 ± 9.643	185.627 ± 45.265	11.563 ± 1.012	10.091 ± 5.322	3.629 ± 0.701	260.520 ± 58.040
CG	17.780 ± 7.322	189.772 ± 54.521	11.595 ± 0.836	10.431 ± 5.766	3.921 ± 0.917	261.321 ± 62.694
GG	18.372 ± 7.864	185.568 ± 41.961	11.569 ± 0.759	11.353 ± 6.254	3.724 ± 0.755	237.510 ± 49.367
P[a]	0.740	0.853	0.970	0.677	0.121	0.051
**miR-631I/D**						
II	18.066 ± 7.996	187.707 ± 49.780	11.576 ± 0.866	10.598 ± 5.648	3.796 ± 0.830	255.700 ± 59.949
ID	20.600 ± 7.905	188.077 ± 46.320	11.633 ± 0.796	9.976 ± 6.517	3.854 ± 0.935	252.067 ± 50.730
P[a]	0.336	0.979	0.788	0.703	0.81	0.820
**miR-938G>A**						
GG	18.345 ± 7.970	186.909 ± 49.650	11.586 ± 0.861	10.456 ± 5.685	3.801 ± 0.844	255.81 ± 59.788
GA	14.333 ± 9.504	216.600 ± 31.214	11.350 ± 0.751	20.000	3.750 ± 0.311	240.400 ± 27.199
P[a]	0.392	0.186	0.588	0.097	0.904	0.567
**miR-1302-3C>T**						
CC	18.000 ± 7.847	189.463 ± 51.042	11.583 ± 0.882	10.427 ± 5.618	3.814 ± 0.851	254.239 ± 59.177
CT	21.800 ± 9.545	174.579 ± 31.964	11.569 ± 0.620	12.354 ± 6.895	3.678 ± 0.721	263.400 ± 60.440
TT	NA	161.000	NA	4.370	3.800	316.000
P[a]	0.169	0.402	0.945	0.334	0.809	0.479

Abbreviations: RPL, recurrent pregnancy loss; T. chol, Total cholesterol; PAI-1, plasminogen activator inhibitor-1; PT, prothrombin time; PLT, platelet count; SD, standard deviation.
[a]One-way analysis of variance test.

Supplementary Table S3

Clinical variables in RPL patients with pregnancy loss ≥3 stratified by microRNA polymorphism status

Genotypes	CD56+ NK Cells (%) Mean ± SD	T. chol (mg/dl) Mean ± SD	PT (sec) Mean ± SD	PAI-1 (ng/ml) Mean ± SD	Uric acid (mg/dl) Mean ± SD	PLT (10^3/ul) Mean ± SD
**miR-604A>G**						
AA	17.425 ± 7.928	189.757 ± 54.029	11.657 ± 0.816	10.795 ± 5.726	3.844 ± 0.855	244.825 ± 57.931
AG	18.203 ± 9.308	191.179 ± 61.182	11.672 ± 0.944	9.958 ± 4.161	3.759 ± 0.818	256.830 ± 51.186
GG	17.625 ± 9.211	181.200 ± 31.247	11.440 ± 0.690	9.617 ± 6.041	4.010 ± 0.886	258.250 ± 38.805
P^a	0.927	0.879	0.738	0.810	0.691	0.525
**miR-608C>G**						
CC	18.695 ± 10.468	178.655 ± 51.508	11.602 ± 0.959	9.397 ± 4.860	3.733 ± 0.568	243.571 ± 49.083
CG	17.418 ± 7.719	199.268 ± 60.245	11.653 ± 0.827	10.826 ± 5.018	4.079 ± 0.976	257.840 ± 58.988
GG	17.677 ± 8.617	183.625 ± 44.412	11.673 ± 0.850	11.593 ± 6.817	3.313 ± 0.515	250.048 ± 40.169
P^a	0.860	0.275	0.952	0.524	0.012	0.511
**miR-631I/D**						
II	17.678 ± 8.707	189.654 ± 56.148	11.668 ± 0.854	10.409 ± 5.154	3.829 ± 0.828	252.739 ± 53.254
ID	19.500 ± 7.969	185.400 ± 34.004	11.338 ± 0.968	9.985 ± 5.800	3.760 ± 1.046	244.429 ± 46.669
P^a	0.621	0.868	0.300	0.852	0.860	0.689
**miR-938G>A**						
GG	17.891 ± 8.584	189.407 ± 54.978	11.643 ± 0.863	10.361 ± 5.172	3.825 ± 0.835	252.152 ± 52.643
GA	14.500 ± 13.435	NA	NA	NA	NA	NA
P^a	0.586	NA	NA	NA	NA	NA
**miR-1302-3C>T**						
CC	17.692 ± 8.453	191.922 ± 57.489	11.670 ± 0.887	9.794 ± 4.830	3.845 ± 0.863	251.261 ± 51.729
CT	19.640 ± 12.015	167.889 ± 11.677	11.438 ± 0.636	17.307 ± 4.572	3.656 ± 0.559	259.273 ± 61.772
TT	NA	NA	NA	NA	NA	NA
P^a	0.627	0.217	0.383	0.004	0.524	0.637

Abbreviations: RPL, recurrent pregnancy loss; T. chol, total cholesterol; PAI-1, plasminogen activator inhibitor-1; PT, prothrombin time; PLT, platelet count; SD, standard deviation. Statistically significant values are indicated in red.

[a] One-way analysis of variance test.

Supplementary Table S4

Plasma folate and homocysteine levels according to miRNA polymorphic genotype in RPL patients with pregnancy loss ≥2 and ≥3

	**PL≥2**	**PL≥3**	**PL≥2**	**PL≥3**
**Genotypes**	**Folate (mg/ml)**		**Hcy (mmol/L)**	
**miR-604A>G**				
AA	14.604 ± 15.832	13.973 ± 16.770	7.136 ± 2.414	7.198 ± 2.152
AG	13.579 ± 8.221	14.487 ± 9.712	6.881 ± 1.710	6.981 ± 1.802
GG	15.709 ± 9.085	14.948 ± 9.217	6.744 ± 2.367	6.370 ± 1.693
P^a	0.690	0.964	0.523	0.323
**miR-608C>G**				
CC	12.771 ± 8.192	12.868 ± 8.964	7.375 ± 2.651	7.241 ± 2.216
CG	14.142 ± 12.870	15.025 ± 15.841	6.855 ± 1.847	7.045 ± 1.932
GG	15.593 ± 12.845	14.568 ± 10.727	6.843 ± 1.953	6.720 ± 1.683
P^a	0.469	0.746	0.202	0.472
**miR-631I/D**				
II	14.551 ± 12.274	14.817 ± 13.326	6.945 ± 2.133	6.971 ± 1.948
ID	10.092 ± 5.317	8.106 ± 1.857	7.330 ± 1.578	7.741 ± 1.975
P^a	0.139	0.135	0.409	0.228
**miR-938G>A**				
GG	14.291 ± 12.021	14.450 ± 13.019	6.961 ± 2.091	6.981 ± 1.935
GA	9.625 ± 3.686	6.610 ± 0.693	7.947 ± 2.506	10.000 ± 0.566
P^a	0.440	0.398	0.351	**0.029**
**miR-1302-3C>T**				
CC	14.110 ± 11.892	14.651 ± 12.848	6.989 ± 2.155	7.004 ± 1.986
CT	14.846 ± 12.808	11.563 ± 14.006	6.812 ± 1.426	7.160 ± 1.595
TT	19.120	NA	7.490	NA
P^a	0.885	**0.006**	0.895	0.776

Abbreviations: RPL, recurrent pregnancy loss; Hcy, homocysteine; SD, standard deviation. Statistically significant values are indicated in red.

[a] One-way analysis of variance test.

Supplementary Table S5
Prediction of microRNA target genes

microRNA	Target gene	Protein product
**miR-604**	MTHFR	Methylenetetrahydrofolate reductase
**miR-608**	IGFBP5	insulin-like growth factor binding protein 5
	EIF5A	eukaryotic translation initiation factor 5A
	TNFAIP1	tumor necrosis factor, alpha-induced protein 1
**miR-631**	TGFB3	transforming growth factor, beta 3
	TIMP2	TIMP metallopeptidase inhibitor 2
	IGF1R	insulin-like growth factor 1 receptor
**miR-938**	GNRHR	gonadotropin-releasing hormone receptor
	TCF7L2	transcription factor 7-like 2 (T-cell specific, HMG-box)
**miR-1302-3**	TRAF4	TNF receptor-associated factor 4

Supplementary Table S6
Location & host gene name of miRNA

miRNA name	Location(chromosome)	host gene
miR-604	10p11.23	SVIL-203(intron 9), Supervillin
miR-608	10q24.31	SEMA4G(intron 3), Semaphorin-4G
miR-631	15q24.2	NEIL1(intron 5), Endonuclease VIII-like 1
miR-938	10p11.23	SVIL-201(intron 3), Supervillin
miR-1302-3	2q13	intergenic

Supplementary Table S7
Primer, PCR condition, enzyme information for PCR-RFLP

miRNA	primers
miR-604	F:CTT GGC TCA GTG GTC TGT TT
	R:GTA CAG GGA CTG AAA GGT GAA G
miR-608	F:GTG GGT CAC ACT TGT AAT CT
	R:AAT TCT GAG GGT GTT CAC TG
miR-631	AAT CCC ACT CCA GGA TGG GAA A
	TGA CAG AGG AAC AGG CAG AGA T
miR-938	T GGT GCA CTG GGT TCA CCT TTA AGC G
	GTA ATA CCT CTG AGC CTT TGG GGC C
miR-1302-3	AAC TAA GCT TGG GAA ATA TTT ATG CCA
	GAG CAT CAT CAG TCC AAA GTC C

PCR condition	
initial denaturation	95°C for 5 min
denaturation	95°C for 30 sec
annealing	59°C for 30 sec
extension	72°C for 30 sec
final extension	72°C for 5 min
total cycles	35 cycles

restriction enzymes

miR-604	*Bss*SI
miR-608	*Pvu*II
miR-631	*Nla*IV
miR-938	*Hha*I
miR-1302-3	*Nla*III

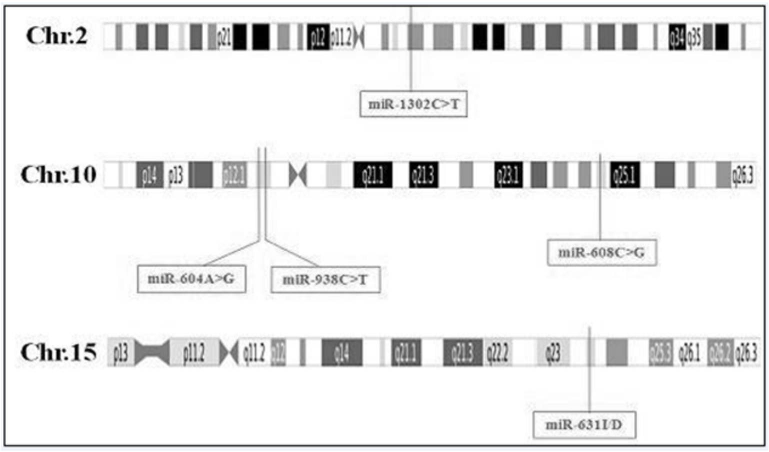

Figure S1: The respective loci of the five miRNAs analyzed in this study (miR-604/miR-608/miR-631/miR-938/miR-1302-3), present on chromosomes 2, 10, and 15.

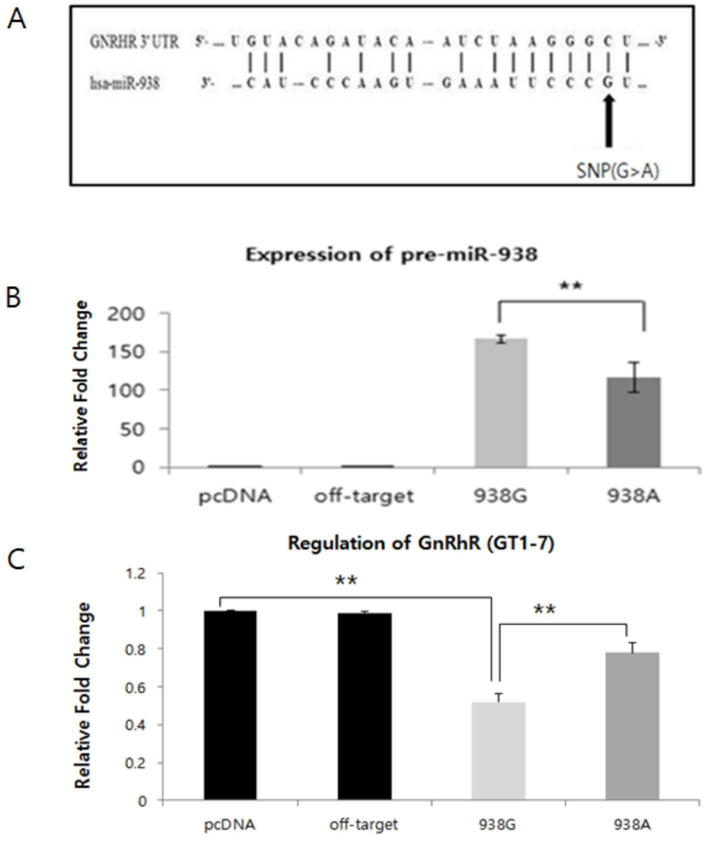

Figure S2. The miRNA, miR-938, regulates GnRHR mRNA via a targeting sequence located in the 3′ UTR of this gene. A. A schematic representation of putative target genes with 3′-UTRs that contain possible miR-938-G and miR-938-A binding sites in conserved regions. B. Expression of miR-938G>A. Levels of miR-938 were detected by miRNA reverse transcriptase quantitative PCR (qRT-PCR) in cells transfected with the empty pCR3.1 vector, pCR3.1-miR-offtarget, pCR3.1-miR-938-GG, or pCR3.1-miR-938-AA. U6 snRNA was used as an internal control, with the relative level of miR-938 normalized to U6. **P<0.05. C. Dual-luciferase reporter assays to measure the strength of interaction between miR-938 and its target sequence in the GnRHR 3′UTR. Reporter constructs contain the predicted targeting sequence for the GnRHR gene (pGL4.13-GnRHR 3′UTR) cloned into the 3′UTR of

the reporter gene. Data represent three independent experiments with triplicate measurements. **$P < 0.05$.
